# Massive bowel resection modulates the expression of genes involved in lipid and cholesterol metabolism in rats

**DOI:** 10.17912/micropub.biology.001253

**Published:** 2024-09-06

**Authors:** Taku Hebiguchi, Mayako Morii, Ryo Watanabe, Hiroaki Yoshino, Yoshihiro Mezaki

**Affiliations:** 1 Department of Pediatric Surgery, Akita University Graduate School of Medicine, Akita-city, Akita, Japan; 2 Department of Pediatric Surgery, Akita Kousei Medical Center, Akita-city, Akita, Japan; 3 Department of Pediatric Surgery, Akita Red Cross Hospital, Akita-city, Akita, Japan; 4 Department of Laboratory Medicine, Jikei University Graduate School of Medicine, Minato-ku, Tokyo, Japan.

## Abstract

We have previously shown that vitamin A-absorptive function was enhanced in bowel-resected rats via increased expression of cellular retinol-binding protein II (CRBP II). Recently, CRBP II was shown to bind not only to retinol but also to monoacylglycerols to modulate gut endocrine signaling. We hypothesized that the increased CRBP II in bowel-resected rats had broader effects than vitamin A metabolism. Acetyl-CoA carboxylase 1 (fatty-acid biosynthesis) and sterol O-acyltransferase 1 (cholesterol esterification) expressions were down-regulated in the bowel-resected rats. Adjustment of nutritional absorption may take place in a limited area of the small intestine by the modulation of gene expression.

**Figure 1. Quantitative analysis of mRNA levels in short bowel (SB) and sham rats:**
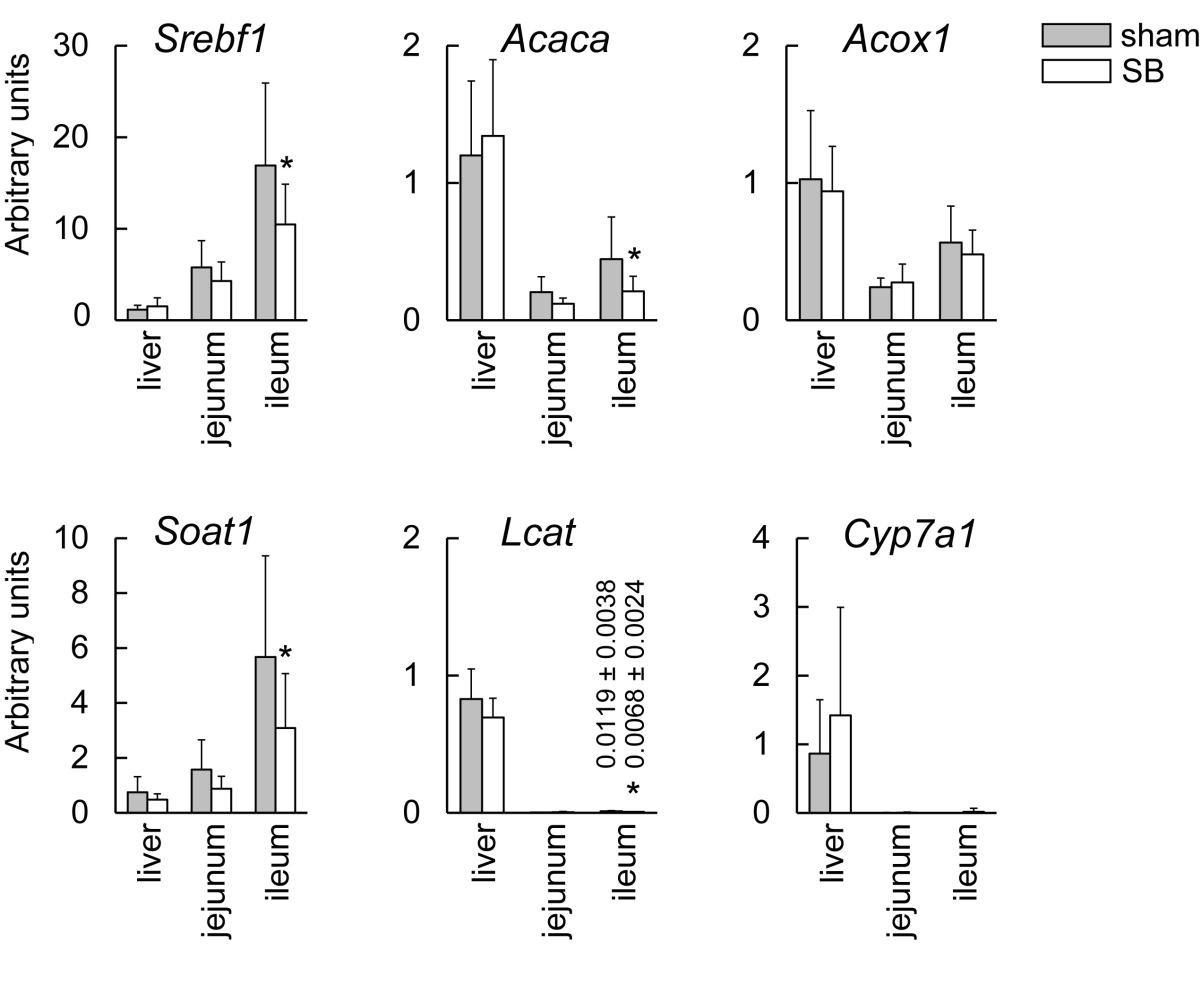
The mRNA levels of sterol regulatory element-binding transcription factor 1 ( *Srebf1* ), acetyl-CoA carboxylase-alpha ( *Acaca* ), acyl-CoA oxidase 1 ( *Acox1* ), sterol O-acyltransferase 1 ( *Soat1* ), lecithin:cholesterol acyltransferase ( *Lcat* ), and cholesterol 7-alpha-hydroxylase 1 ( *Cyp7a1* ) were assessed by quantitative RT-PCR. Data are expressed as the means ± SD. *P < 0.05 was considered to indicate a statistically significant difference.

## Description

In humans, short bowel (SB) syndrome is characterized by a significant reduction in the absorptive surface area of the intestine due to congenital or acquired disorders of the newborn (Belza and Wales 2022) . Intestinal adaptation is a compensatory process that involves various structural and functional alterations to enhance nutritional absorption by the enterocytes. We and others have previously shown that mRNAs for cellular retinol-binding protein II (CRBP II, gene symbol *Rbp2* ) and apolipoprotein A-IV (apoA4) are upregulated in the small intestine after massive bowel resection in rats (Takase et al., 1993; Rubin et al., 1996; Hebiguchi et al., 2015) . CRBP II binds to retinol and retinal and delivers these compounds to retinaldehyde reductases or lecithin:retinol acyltransferase to synthesize retinyl esters, which are then incorporated in chylomicrons (Harrison 2012) . ApoA4 is a component of chylomicrons that enhances lipid transport in enterocytes (Lu et al., 2002) . Given these findings, we proposed that vitamin A-absorptive function is enhanced in the small intestine of bowel-resected rats via enhancement of vitamin A esterification and chylomicron formation (Hebiguchi et al., 2015) .

Recently, CRBP II was shown to bind not only to retinol and retinal but also to monoacylglycerols (MAGs) such as 2-arachidonoylglycerol (2-AG), 1-AG, 2-oleoylglycerol, and 2-lineoylglycerol (Lee et al., 2020) . It was shown in the same report that male *Rbp2* ^-/-^ mice gained more body fat than littermate controls, probably due to increased intestinal MAG concentrations to facilitate enteroendocrine cell release of glucose-dependent insulinotropic polypeptide and subsequent fat cell uptake of fatty acids from circulation (Lee et al., 2020) . We hypothesized that the CRBP II-induction in bowel-resected rats had broader effects on the metabolism of not only vitamin A but also other nutritional components such as lipids and cholesterol. Specifically, upregulation of *Rbp2* mRNA in SB rats may sequester MAGs from functioning as a signaling molecule to modulate expression of genes concerning lipids and cholesterol metabolism in the intestine (Izzo and Sharkey 2010) . To examine this hypothesis, we re-evaluated the expression levels of genes related to lipid and cholesterol metabolism in the livers and intestines of bowel-resected rats, as done previously (Hebiguchi et al., 2015) .

Ileal expression levels of *Acaca* mRNA in SB rats were significantly lower than in shams ( Fig. 1 ). Acetyl-CoA carboxylase-alpha, the protein product of *Acaca* , provides malonyl CoA as a substrate for fatty acid synthase and is the rate-controlling enzyme of fatty acid biosynthesis (Brown and Goldstein 1997) . Expression levels of *Srebf1* mRNA in SB ileum were also significantly lower than in shams ( Fig. 1 ). The *Srebf1* gene encodes sterol regulatory element-binding protein (SREBP)-1, a membrane-bound transcription factor that stimulates *Acaca* gene transcription (Lopez et al., 1996) . Given these observations, it is suggested that fatty-acid biosynthesis was down-regulated in the ileum of SB rats. Acyl-CoA oxidase 1 is a peroxisomal enzyme that initiates fatty acid beta-oxidation (Varanasi et al., 1994) . The expression levels of *Acox1* mRNA in the liver and intestine were unchanged in SB and sham rats ( Fig. 1 ), implying that fatty acid catabolism was unchanged. Takase and her colleagues found that jejunal expression levels of *Rbp2* were upregulated by dietary fats, especially unsaturated fatty acids (Goda et al., 1994; Suruga et al., 1995) . However, the current study did not analyze the cause-and-effect relationships between *Rbp2* mRNA upregulation and reduced fatty-acid biosynthesis in SB rat intestines.

Sterol O-acyltransferase 1 (synonym: acyl-CoA:cholesterol acyltransferase 1, ACAT1), the product of *Soat1* , is an intracellular protein that esterifies cholesterol (Cadigan et al., 1988) . Molina et al. reported that sterol O-acyltransferase activity was decreased in the livers of SB rats at six weeks after surgery but did not show any enzymatic activity in the intestine (Molina et al., 1989) . In the current study using SB rats at one week after surgery, no difference was observed in hepatic expression levels of *Soat1* mRNA between SB and sham rats, whereas ileal expression levels of *Soat1* mRNA were significantly lower in SB rats than in shams ( Fig. 1 ). Intestinal sterol O-acyltransferase activity is thought to be important for the absorption of cholesterol (Wilson and Rudel 1994) . Our results suggested that cholesterol absorption was decreased in the ileum of SB rats. The other cholesterol-esterifying enzyme, lecithin:cholesterol acyltransferase (LCAT), is a secretory enzyme that esterifies cholesterol in plasma (Pavanello and Calabresi 2020) . The expression levels of *Lcat* mRNA were unchanged in the livers between SB and sham rats but were significantly lower in the ileum of SB rats. However, the expression levels in the intestines were very low compared to those in the liver ( Fig. 1 ). It was not studied whether the reduced cholesterol absorption and increased vitamin A absorption affected the constituents of the chylomicron, which contains both cholesteryl esters and retinyl esters.

*Cyp7a1* mRNA, the gene for the rate-limiting enzyme of bile acid synthesis, is upregulated when enterohepatic circulation is insufficient to compensate for the loss of bile salts through the intestine (Ferrebee and Dawson 2015) . In addition, oral administration of vitamin A reduces the expression levels of *Cyp7a1* mRNA (Schmidt et al., 2010) , suggesting that induced intestinal absorption of vitamin A in SB rats might decrease the hepatic expression levels of *Cyp7a1* mRNA. In the current study, however, hepatic expression levels of *Cyp7a1* mRNA did not differ between SB and sham rats ( Fig. 1 ), implying that the summation of these effects might negate a requirement for altered gene expression.

Our current results and those of previous reports (Hebiguchi et al., 2015) indicate that fatty-acid biosynthesis and cholesterol-esterification are down-regulated in bowel-resected rats whereas vitamin A absorption is upregulated in the ileum of SB rats. It should be noted that saturated fatty acids and cholesterol can be synthesized in the body, whereas vitamin A must be taken exclusively from food. The absorptive area in SB rat intestines is limited; therefore fine-tuning of nutritional absorption may be necessary to meet the nutritional requirements of SB animals. In our experiments, it was not analyzed whether the diminished *Srebf1* , *Acaca* and *Soat1* levels in the ileum of SB rats affect absorption and subsequent plasma levels of fatty acid, triglyceride and cholesterol ester. Therefore, it should be noted that our conclusion is preliminary and needs further verification.

## Methods

**Animals and surgical procedures**

Protocols for animal experimentation were approved by the Animal Research Committee, Akita University Graduate School of Medicine. All animal experiments adhered to the “Guidelines for Animal Experimentation” of the university. The experimental procedures for generating 75% bowel-resected rats were described previously (Hebiguchi et al., 2015) . Outbred male Sprague-Dawley rats Slc:SD (RRID:RGD_12910483) (Japan SLC, Hamamatsu, Japan) were used in this study. In SB rats, the gut was resected from a point 5 cm distal to the ligament of Treitz to a point 10 cm proximal to the ileocecal junction. In sham rats, the gut was divided 10 cm proximal from the ileocecal junction and anastomosed without resecting any part. The rats were fasted for 24 h with free access to water and then fed with Elental (Ajinomoto Pharmaceuticals, Tokyo, Japan). Samples were collected 7 days after the surgery. A pair of rats (one for SB and the other for sham operation) underwent surgery for each trial. From the 19 pairs of rats, 13 SB and 14 sham-operated rats completed the trial. The samples were collected from all these animals, but the jejuna of the sham-operated rats were collected from 8 animals out of the 14 animals that completed the trial.

**RNA extraction and quantitative RT-PCR**

RNA extraction and quantitative RT-PCR were done as described previously (Hebiguchi et al., 2015) . We did not repeat quantitative PCR from the same sample for each gene, and only variations of biological replicates were evaluated.

**Statistical analysis**

Data were expressed as means ± standard deviation (SD). The statistical significance of differences was evaluated by ANOVA combined with the Tukey test. P values of < 0.05 were considered to be statistically significant.

## Reagents

**Table:** 

**Primer Name**	**Sequence (5' to 3')**
*Gapdh* -forward	ACAGCAACTCCCATTCTTCC
*Gapdh* -reverse	TCCACCACCCTGTTGCTGTA
*Srebf1* -forward	CAGCTCCTGGACCGCAGT
*Srebf1* -reverse	AGCAGGAGGCCAACAGCAA
*Acaca* -forward	CGCTCTGTGATAGAGGAGAA
*Acaca* -reverse	TGTGCTGGGTCATGTGGAC
*Acox1* -forward	CTTGGCCGCTATGATGGAAA
*Acox1* -reverse	CAAAGCTTGGACTGCAGGG
*Soat1* -forward	GCCAAGAAGAGGCAGTTGG
*Soat1* -reverse	CCATTGTCCAGAGATGCAGAC
*Lcat* -forward	GGTTCCATCAAGCCCATGC
*Lcat* -reverse	TGAGCTGGAAACATCCAGGG
*Cyp7a1* -forward	GTTCCTATAACATCCGAAAAGA
*Cyp7a1* -reverse	GGTACCTTGATGAAAGCGGGAAAG
